# Hypertrophy of Rat Skeletal Muscle Is Associated with Increased SIRT1/Akt/mTOR/S6 and Suppressed Sestrin2/SIRT3/FOXO1 Levels

**DOI:** 10.3390/ijms22147588

**Published:** 2021-07-15

**Authors:** Zoltan Gombos, Erika Koltai, Ferenc Torma, Peter Bakonyi, Attila Kolonics, Dora Aczel, Tamas Ditroi, Peter Nagy, Takuji Kawamura, Zsolt Radak

**Affiliations:** 1 Research Center of Molecular Exercise Science, University of Physical Education, H-1123 Budapest, Hungary; gzoltan5@gmail.com (Z.G.); koltai.erika@tf.hu (E.K.); torma.ferenc@tf.hu (F.T.); bakonyi.peti@gmail.com (P.B.); kolonics.attila@tf.hu (A.K.); aczel.dora555@gamil.com (D.A.); 2 Department of Molecular Immunology and Toxicology, National Institute of Oncology, H-1122 Budapest, Hungary; tamas.ditroi@oncol.hu (T.D.); peter.nagy@oncol.hu (P.N.); 3 Department of Anatomy and Histology, University of Veterinary Medicine, H-1078 Budapest, Hungary; 4 Faculty of Sport Sciences, Waseda University, Tokorozawa 2-579-15, Japan; tkawamura@aoni.waseda.jp

**Keywords:** overload-induced hypertrophy, skeletal muscle, anabolic signaling pathways, redox regulation

## Abstract

Despite the intensive investigation of the molecular mechanism of skeletal muscle hypertrophy, the underlying signaling processes are not completely understood. Therefore, we used an overload model, in which the main synergist muscles (gastrocnemius, soleus) of the plantaris muscle were surgically removed, to cause a significant overload in the remaining plantaris muscle of 8-month-old Wistar male rats. SIRT1-associated pro-anabolic, pro-catabolic molecular signaling pathways, NAD and H_2_S levels of this overload-induced hypertrophy were studied. Fourteen days of overload resulted in a significant 43% (*p* < 0.01) increase in the mass of plantaris muscle compared to sham operated animals. Cystathionine-β-synthase (CBS) activities and bioavailable H_2_S levels were not modified by overload. On the other hand, overload-induced hypertrophy of skeletal muscle was associated with increased SIRT1 (*p* < 0.01), Akt (*p* < 0.01), mTOR, S6 (*p* < 0.01) and suppressed sestrin 2 levels (*p* < 0.01), which are mostly responsible for anabolic signaling. Decreased FOXO1 and SIRT3 signaling (*p* < 0.01) suggest downregulation of protein breakdown and mitophagy. Decreased levels of NAD^+^, sestrin2, OGG1 (*p* < 0.01) indicate that the redox milieu of skeletal muscle after 14 days of overloading is reduced. The present investigation revealed novel cellular interactions that regulate anabolic and catabolic processes in the hypertrophy of skeletal muscle.

## 1. Introduction

Atrophy of skeletal muscle could be a consequence of exposure to anti-gravitation, immobilization, cancer therapy, or aging [1,2,3], with serious functional and pathophysiological outcomes [4]. On the other hand, hypertrophy of skeletal muscle has benefits on health and sport performance as well [5]. Hence, as a result of intensive investigation a lot is known about the molecular pathways that are involved in increased protein synthesis and attenuated catabolic processes, which happen to occur during the hypertrophy of skeletal muscle [6,7]. One of the well accepted models of muscle hypertrophy on rodents is the overload-induced hypertrophy in which the surgical removal of gastrocnemius and soleus muscles results in a 30–40% increase in the mass of plantaris muscle [8,9,10]. Recently, we discovered that the NAD-dependent histone deacetylase SIRT1 is upregulated during muscle hypertrophy and associated with enhanced nicotinamide phosphoribosyltransferase (NAMPT), Akt, endothelial nitric oxide synthase (eNOS), and glucose transporter type 4 (GLUT4) levels, and suppressed forkhead box class O protein 1 (FOXO1) [8]. However, that study, just examined SIRT1-associated cellular pathways, and important regulatory proteins, like Akt and mTOR are not studied in details. The Akt-mediated cellular pathways promote cellular survival by supporting proliferation and inhibiting apoptosis [11]. The protein kinase called mechanistic target of rapamycin (mTOR) is a downstream regulator of Akt and stimulates protein synthesis via ribosomal protein S6 kinase (S6 kinase) [12]. The mTOR/Akt pathway is upregulated during muscle hypertrophy and down regulated at atrophy [13]. The adenosine monophosphate activated protein kinase (AMPK) signaling, which is activated upon energy depletion, such as exercise or caloric restriction, could curb the activation of mTOR signaling [14].

Sestrins are highly conserved but functionally not well characterized p53 modulated proteins with antioxidant activity [15], which can inhibit mTOR via AMPK [16]. Although sestrin and SIRT1 are distinct proteins, the fact that SIRT1 deacetylates p53 and sestrins are regulated by p53 might link them functionally. However, this possible link needs to be investigated. One of the intrinsic activators of SIRT1 is H_2_S [17] and this gas has antioxidant effects, suppresses oxidative stress [17,18,19] and, hence, modifies the NAD/NADH ratio which can lead to increased SIRT1 activity [20,21]. Therefore, based on these characteristics of SIRT1, sestrin and H_2_S it cannot be excluded that they could be involved of the regulation of muscle hypertrophy. Therefore, we tested whether newly discovered role of SIRT1 in muscle hypertrophy involves modulation of mTOR, S6, sestrin, and H_2_S producing proteins.

## 2. Results

**Figure 1:**
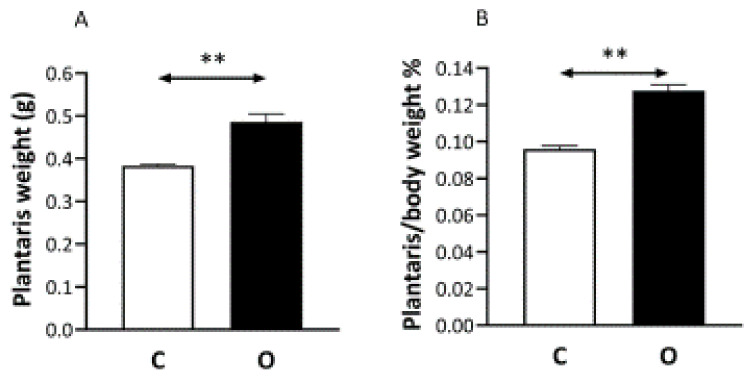
The effects of overload on muscle mass. The removal of gastrocnemius and soles muscles resulted in greater weight carrying load on the plantaris muscle, which significantly increased in the muscle mass of plantaris (O) compared to control (C) muscle (Panel (**A**)). Panel (**B**) shows the muscle in a relation to body mass is expressed. *n* = 9, ** *p* < 0.01, Results are expressed as mean ± SE.

Fourteen days after surgery the weight of the plantaris muscle increased by 43% (Figure 1A). Plantaris/body weight ratio also changed significantly (Figure 1B).

**Figure 2:**
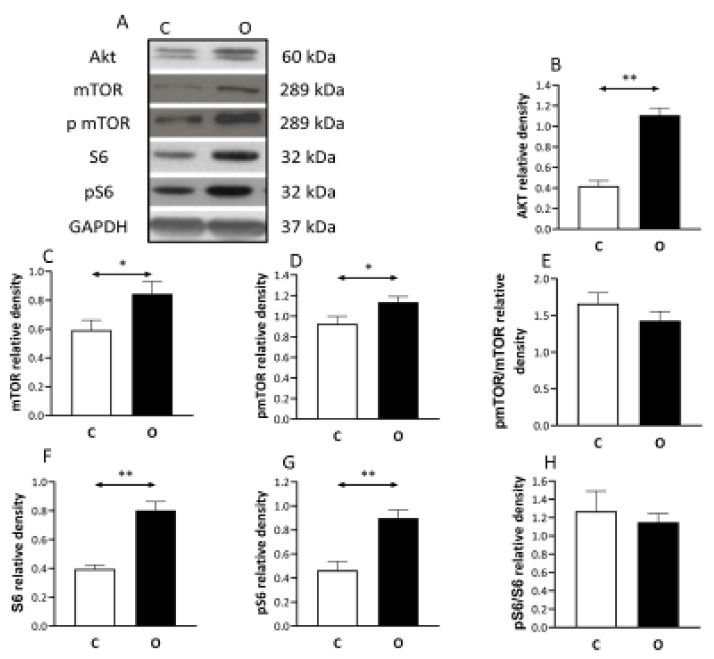
Pro anabolic pathway during muscle hypertrophy. Overload induce muscle hypertrophy increases the contents of pro-anabolic Akt, mechanistic target of rapamycin mTOR (mTOR) and ribosomal protein S6 kinase (S6 kinase) compared to control muscle, suggesting the significant involvement of this signaling pathway. (**A**) panel shows the selected immunoplot signals, and (**B**–**H**) panels show the densitometric results of nine samples in each group. C: control group, O: Overloaded group, *n* = 9, * *p* <0.05, ** *p* < 0.01, Results are expressed as mean ± SE.

The level of the anabolic factors increased in the overload group. The overload increased the level of the Akt, mTOR, pmTOR, S6, pS6 proteins significantly (Figure 2).

**Figure 3:**
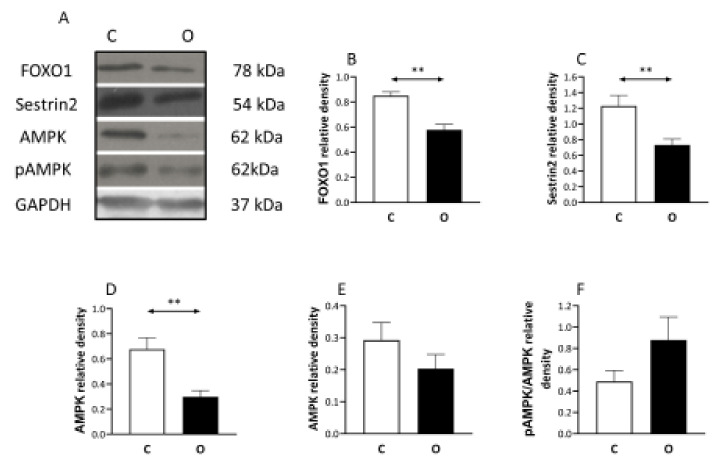
Pro-catabolic pathway associated proteins are down-regulated during overload. Because hypertrophy is not just dependent on anabolic but also on catabolic signaling, the level of forkhead box class O protein 1 (FOXO1), Sestrin 2, AMP, pAMPK, were measured and found to be decreased in the muscle which is exposed to overload-induced hypertrophy, indicating the down-regulation of catabolic pathways during overload-induced hypertrophy. (**A**) panel shows the selected immunoplot signals, and (**B**–**F**) panels show the densitometric results of nine samples in each group. C: control group, O: Overloaded group, *n* = 9, ** *p* < 0.01, Results are expressed as mean ± SE.

On the other hand, the level of the catabolic protein FOXO1 decreased in the overload group (Figure 3). The Sestrin 2 protein (Figure 3) which negatively regulates the TORC1 signaling pathway showed significant reduction in the operated group. Moreover, the AMPK which is a marker of the cell’s energetic condition showed a significant decrease in the operated group (Figure 3), otherwise pAMPK level did not change.

**Figure 4:**
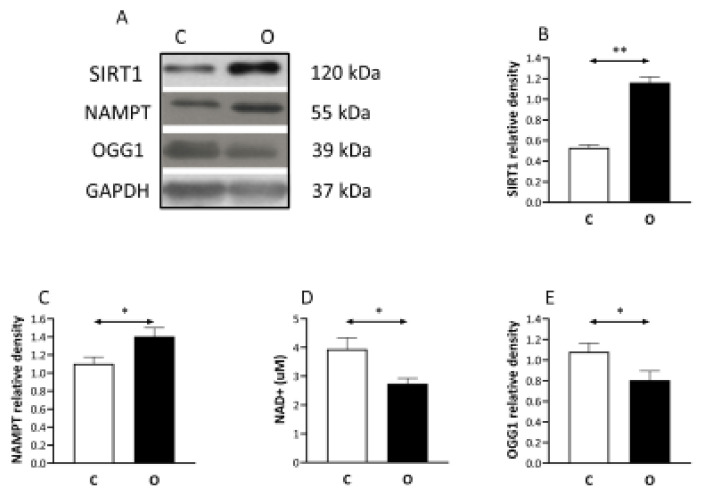
SIRT1 induced by muscle hypertrophy but OGG1 is suppressed. SIRT1 is a NAD^+^ dependent histone deacetylase, therefore we measured nicotinamide phosphoribosyltransferase (NAMPT) which is a rate-limiting enzyme in the NAD^+^ salvage pathway. The levels of SIRT1, NAMPT increased, while NAD^+^ contents decreased as a result of muscle hypertrophy, suggesting a regulatory role of SIRT1 in overload induced hypertrophy. (**A**) panel shows the selected immunoplot signals, and (**B**–**E**) panels show the densitometric results of nine samples in each group. C: control group, O: Overloaded group, *n* = 9, * *p* <0.05, ** *p* < 0.01, Results are expressed as mean ± SE.

The level of SIRT1 (Figure 4), and NAMPT elevated significantly in the operated group. The activity of NAD (Figure 4), as well as the content of DNA repair enzyme of OGG1 levels was significantly lower in the operated group than in the control.

**Figure 5:**
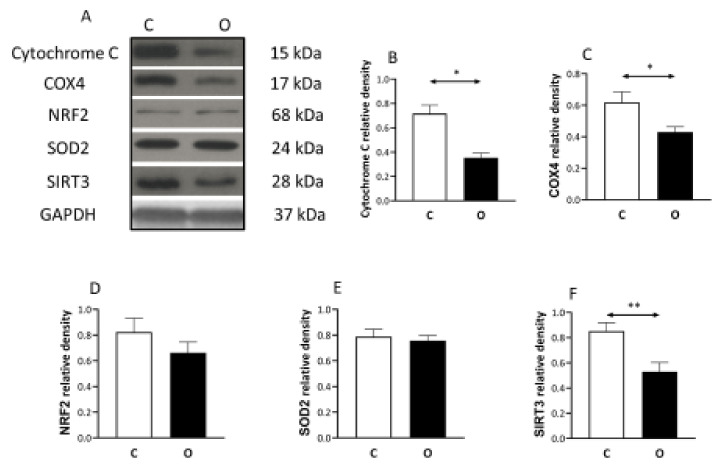
The levels of mitochondrial proteins in hypertrophied muscle. The levels of mitochondrial related proteins suggest that the increase in muscle mass was not associated with the increase in mitochondrial proteins with the same degree during overload-induced hypertrophy. (**A**) panel shows the selected immunoplot signals, and (**B**–**F**) panels show the densitometric results of nine samples in each group. C: control group, O: Overloaded group, *n* = 9, * *p* <0.05, ** *p* < 0.01, Results are expressed as mean ± SE.

It seems that the increased muscle size was not associated with similar increase in mitochondrial content, judged by the levels of Cytochrome C, COX4, SOD2, SIRT3 decreased significantly in the overload group compared to the control, while the decrease in Nrf2 protein concentration did not reach the significant levels (Figure 5).

**Figure 6:**
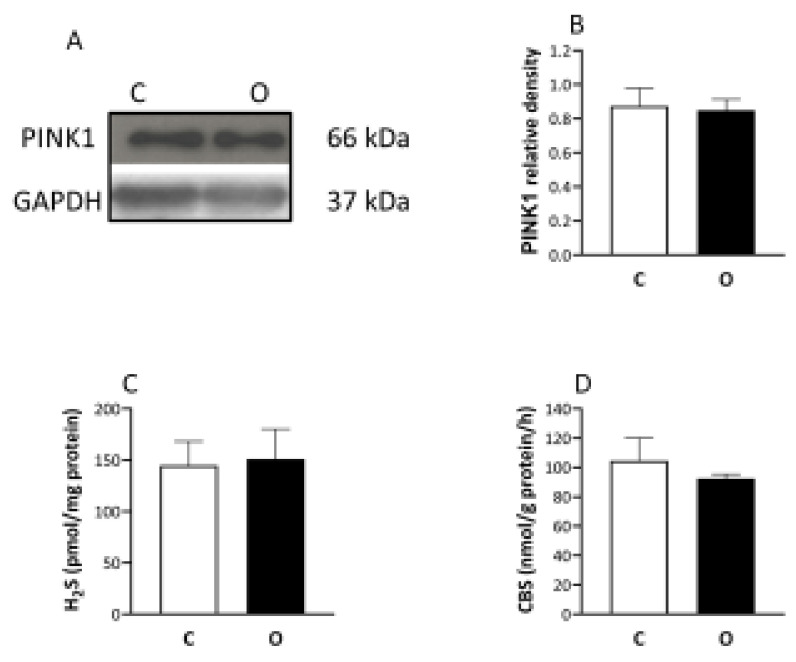
The levels of PINK1, H2S, and CBS did not change the muscle hypertrophy. The unaltered levels of PINK1 and H_2_S, as well as cystathionine-β-synthase (CBS), rules out the significant modulating role of mitophagy or H_2_S contribution through activation of SIRT1 in increased muscle mass. (**A**) panel shows the selected immunoplot signals, and (**B**–**D**) panels show the densitometric results of nine samples in each group. C: control group, O: Overloaded group, *n* = 9, Results are expressed as mean ± SE.

Finally, the mitophagy marker of PINK1 did not change after the operation (Figure 6), and the level of monobromobimane measured H_2_S and the activity of the cystathionine-β-synthase (CBS) enzyme (Figure 6), which is one of the enzymes responsible for the hydrogen sulfide formation, did not show any difference between the groups.

## 3. Discussion

Additionally, the confirmation of the involvement of SIRT1 in overload induced hypertrophy the novel observations of this study revealed that the SIRT1- mediated pathways include the activation of mTOR and S6 proteins. Moreover, we have discovered novel mosaics of the complex cellular regulation of muscle hypertrophy.

One of the major roles of p53, which is a powerful tumor suppressor, is to inhibit cell proliferation while cell growth is positively regulated by mTOR [16]. It has been shown that Sestrin2, which is a highly conserved protein and target of p53, activates AMPK which can lead to inhibition of mTOR [16]. Independently from the inhibitory role of Sestrin2 on mTOR, this protein is an antioxidant since it acts as a cysteine reductase and modulates peroxide signaling [15]. In the present overload-induced hypertrophy model, we have found increased SIRT1, Akt, mTOR, and S6 levels which were associated with decreased protein levels of Sestrin2 and AMPK. The phosphorylation ratio of AMPK did not change significantly, but the decreased protein levels suggest that the cellular adaptation either decreased the synthesis or increased the degradation of AMPK to overload induced hypertrophy.

Prolonged activation of mTOR could generate ROS and activate sestrins [14]. However, this could be not the case in the present study, since Sestrin2 or OGG1 levels were decreased in the overload-induced hypertrophy, compared to control muscle. This suggestion is further supported by that fact that Sestrin2s are positive regulators of Nrf2 pathways, most likely due to the antioxidant capacity of Sestrin2 [22]. In the present hypertrophy model, the Sestrin2 level decreased in parallel with Nrf2, although the decrease in Nrf2 case was just a tendency. Moreover, it has also been reported that in cell culture, knockdown of sestrin2 reduced AMPK and SOD2 levels [23], and we have observed simultaneous effect during overload induced hypertrophy. In addition, we measured decreased level of SIRT3, the enzyme which deacetylates two critical lysine residues on SOD2, promotes its antioxidant activity, and decreases the level of ROS in the mitochondria [24]. Because NAD^+^ levels of overloaded muscle were lower than those of controls, it is suggested that in hypertrophied skeletal muscle at the time of sampling there was a reduced cellular milieu.

In addition, it has been shown that sestrins play critical roles in exercise-induced adaptation since sestrins are required to increase endurance, insulin sensitivity and mitochondrial biogenesis via PGC-1 alpha [25]. Interestingly, in our overload-induced hypertrophy, the decreased sestrin2 levels were associated with decreased levels of mitochondrial markers like Cytochrome C, COX4, NRF2, SIRT3. According to our suggestion, the increase in the mass of muscle filaments due to overload was not associated with a similar increase in mitochondrial mass, this could lead to this result. Indeed, a recent paper reports that 14 days of functional overload increased the levels of proteins, which regulate mitochondrial fusion and decreased fission controlling proteins, and this could explain the relative reduction in mitochondrial proteins [26].

In this study, we have confirmed that SIRT1 levels increased in overload-induced hypertrophy but the possible relationship between sestrin2 and SIRT1 is not well known. It has been shown that resveratrol administration, which activates SIRT1, upregulated the expression of sestrin2 [27]. In another experimental model amyloid beta-induced stress in human neuroblastoma cells showed increased sestrin2 and deceased SIRT1 expression [28]. When the sestrin2 and SIRT1 levels were measured in serum samples of asthma patients, only sestrin2 levels increased compared to control groups [29]. Aging results in decreased sestrin concentration in human skeletal muscle [30]. In a recent study, the effects of daily protein supplementation were measured in downstream responsiveness of skeletal muscle mTOR in human immobilization [31]. It turned out that immobilization reduced postabsorptive skeletal muscle phosphorylation of the mTOR, S6, and sestrin2 [31], suggesting complex regulation and role of sestrin2.

SIRT1 is generally considered to be a protein which increases cell survival [32], and during caloric restriction (CR) the activity and protein levels of SIRT1 are increased [33]. It has been also reported that CR-induced SIRT1 activation is associated with enhanced generation of the small signaling molecule, H_2_S [34]. Since, in our previous study, we have found that overload-induced hypertrophy of skeletal muscle increased the activity and protein levels of SIRT1 [8], which is confirmed in the current study. We measured the activity of CBS, which is one of the major H_2_S producing enzymes [35]. The results of a recent study suggest that exogenous H_2_S (Na H_2_S) injection increased the diameter of fast twitch muscles via activation of mTOR, the S6 pathway leading to increased protein synthesis [36]. H_2_S causes the persulfidation of SIRT1, which increases SIRT1 binding to zinc ion by which the SIRT1 deacetylase activity is increased [37]. However, in our overload induced hypertrophy we could not detect increased levels of bioavailable H_2_S or CBS activity, suggesting that SIRT1 activation has different signaling pathways during CR and hypertrophy. Indeed, CR suppresses mTOR signaling [38], while overload-induced hypertrophy increases mTOR signaling, but in both situations SIRT1 level is increased.

Hypertrophy, the increased protein synthesis of skeletal muscle is regulated by anabolic and catabolic cellular processes. FOXO1 regulates protein breakdown and mitochondrial turnover [39]. Akt can phosphorylate FOXO1, which translocates into the nucleus and thereafter translocates into the cytosol or is degraded [39]. Moreover, SIRT1 can directly deacetylate FOXO1 and decrease the activity of this protein [21]. The decreased levels of FOX1 in hypertrophied muscle, could mean suppressed degradation of proteins [8], however we were also interested in mitochondrial quality control during hypertrophy. Therefore, we measured the content of PINK1, since PINk1 signaling pathway regulates mitochondrial fission, and ubiquitylation, during mitophagy [40]. When PINK1 is activated by loss of the mitochondrial membrane potential or excessive production of ROS, this can readily lead to mitochondrial degradation via phosphorylation of Parkin. Our data from the maintained level of PINK1 suggest that overload-induced hypertrophy does not cause mitochondrial dysfunction. Indeed, we also found decreased levels of SIRT3 in overloaded muscle compared to control, and SIRT3 is implicated in mitophagy, since, in human glioma cells, silencing of SIRT3 blunted the degradation of mitochondria [41]. Therefore, the decreased levels of mitochondrial proteins in overload-induced hypertrophy are unlikely due to enhanced mitophagy, but could be due to the decreased response to mitochondrial biogenesis to overload-induced hypertrophy.

## 4. Methods

### 4.1. Animals

Eighteen middle aged (8 months) male Wistar rats were randomly divided into a control (C) and a hypertrophied (H) group. Animals were held in a thermoneutral room on a 12:12 h photoperiod and were provided with food and water ad libitum. The entire experiment was carried out in the Research Center for Molecular Exercise Science, University of Physical Education of Hungary and approved by the National Ethical Committee (63/2/2017 and PE/EA/62-2/2021).

### 4.2. Synergist Muscle Ablation

The main synergist muscles (*gastrocnemius*, *soleus*) of the plantaris muscle were surgically removed. All the operations were carried out under deep anaesthetic conditions with pentobarbital sodium (50 mg/kg). The surgical procedures were performed bilaterally as described previously [8]. The neural and vascular supplies of the plantaris muscle remained intact. The control group underwent a sham operation when the tendon of the plantaris and its synergist’s tendon were separated carefully but the soleus and the gastrocnemius muscles were not damaged or removed. After the operation and for the next two days the animals were administered analgeticum. The overload period lasted for 14 days and the animals were monitored for the whole period. On day 14 the food was taken away and the next morning the animals were euthanized (decapitation) after an overnight fast. The plantaris muscles were collected immediately after the removal of the fat and the connective tissues. The muscles were weighted and frozen in liquid nitrogen and stored at −80 °C until further analysis.

### 4.3. NAD Measurement

A NAD/NADH Assay Kit (ab176723) was used to measure the NAD levels in the plantaris muscles according to the manufacturer’s instructions. Plantaris muscles were homogenized in NADH/NAD Lysis buffer. Then the samples were centrifuged and separated into treated and untreated parts. The samples and 25 µL diluted NADH standards were loaded into 96-well microplates in duplicate. Then, 25 µL of NAHD/NADH Control Solution was added to the standards and 25 µL of NADH Extraction Solution or NAD Extraction Solution were added, respectively, to the NADH and the NAD samples. After this the plates were heated at 37 °C for 15 min for NAD/NADH decomposition. Then 25 µL of NAD/NADH Control Solution were added to the standards and 25 µL of NAD Extraction Solution or NADH Extraction Solution were added to the NADH and NAD samples, respectively. Then, 75 µL of Reaction Mix were added to all wells. For 2 h the optical density was measured every five min at ex485 and em538 nm wavelength.

### 4.4. Western Blots

The plantaris muscle homogenates were procreated by Ultra Turrax homogenizer (IKA, Staufen im Breisgau, Germany) with 10 vol of lysis puffer. The samples were electrophoresed on 6–15% polyacrylamide (SDS-PAGE) gels. The samples were between 3 and 6 µL. The proteins in the samples were transferred into PVDF membranes. Then, the membranes were blocked with BSA (0.5–5%) or Milk (5%) for 2 h at 4 °C. After blocking the membrane were incubated with primary antibody at 4 °C overnight. Antibody list: SIRT1 1:1000 (Ab:110,304), S6 1:5000 (Cs:2217S), pS6 1:5000 (Cs:5364S), AKT 1:3000 (Cs:46915), mTOR 1:1000 (Cs:29835), p-mTOR 1:1500 (Cs:5536), FOXO1 1:1000 (Cs: 9454), Sestrin2 1:5000, (Ab 23602), AMPK 1:1000 (Cs: 2532), p-AMPK 1:1500 (Cs:2535), NAMPT 1:500 (Ab45,890), Cytochrome C 1:1000 (Sc-7159), COX4 1:2500 (Sc-69,359), Sirt3 1:10,000 (Proteintech:10,099-1-AP), NRF2 1:1000 (Ab:31,163), SOD2 1:3000 (Invitrogen:PA5-80048), PINK1 1:1000 (Affinity: DF7742), OGG1 1:1000 (Proteintech: 15125-1AP), GAPDH 1:3000 (Sigma: G8795). The next day the membranes were washed three-times with Tris-buffered saline-Tween-20 (TBST) at room temperature and incubated with HRP-conjugated secondary antibody for 2 h at 4 °C. After that the membranes were washed again with TBST three times at room temperature. Then the membranes were incubated with chemiluminescent substrate and protein bands were visualized on X-ray films. The bands were quantified by ImageJ software. The relative density was calculated to our housekeeping protein, which was GAPDH.

### 4.5. Measurement of H_2_S with the Monobromobimane Method

H_2_S assay was based on a previously published method adapted here for tissue lysates [42]. First, approximately ~10–20 mg of the tissue samples were disrupted by a dismembrator. Alkylation/lysis was carried out by the addition of 500 µL PBS set to pH 8.0 containing 1 mM monobromobimane (Sigma Aldrich, St. Louis, MO, USA) in a light-protected environment. After a short sonication on ice the solutions were incubated for one hour at 37 °C in the dark. The reaction was stopped by the addition of 50 µL 50% TCA followed by centrifugation at 12,000× *g* 4 °C for 10 min to remove precipitated proteins. Supernatants were removed and transferred into HPLC vials for measurement, and the remaining pellets were redissolved in 300 µL 4% SDS/0.1 M NaOH for BCA protein assay. Bimane labeled species from the supernatants using 3 µL injection volumes were separated on a Phenomenex Luna C18(2) 250 × 2.0 mm × 3μm column on a Thermo Ultimate 3000 UHPLC system (Thermo Fisher, Waltham, MA USA). A linear gradient elution using solvents 0.1% TFA/H_2_O (A) and 0.1% TFA/ACN (B) was carried out as described in Table 1. The fluorescent detector was set to excite at 390 nm and detect emission at 475 nm. Quantitation was conducted by establishing a calibration curve by derivatizing standardized H_2_S solutions.

### 4.6. Measurement of CBS Activity

Frozen tissue samples of ~10–20 mg were disrupted by a dismembrator (B. Braun 853162) followed by the addition of the lysis buffer (150 mM KCl, 50 mM HEPES pH 7.4, 0.1% CHAPS, 2% protease inhibitor cocktail) of 400 µL. After a brief sonication on ice, tubes were placed on a rotator for 30 min at 4 °C. After centrifugation at 12,000× *g*, 4 °C for ten minutes, supernatant protein content was measured by BCA assay. All samples were diluted to 1 mg/mL protein concentration using the lysis buffer. The prepared solutions were used to carry out the CBS activity assay exactly as described previously [43]. In brief, samples were mixed with cofactors (SAM, PLP) and substrates homocysteine (prepared fresh from HCys-thiolactone) and stable isotope labeled serine (2,3,3-d-serine, Cambridge Isotope Laboratories, Inc., Tewksbury, MA, USA) followed by four hours of incubation at 37 °C. Reaction mixtures were quenched using with ”Reagent 1” of the EZ:faast kit (Phenomenex, Torrance, CA, USA) spiked with a known amount of stable isotope labeled cystathionine (3,3,4,4-d-cystathionine, Cambridge Isotope Laboratories, Inc.) as an internal standard. Sample preparation and measurement with the EZ:faast kit was carried out following the manufacturers manual. For the HPLC-MS/MS measurements a Thermo Vanquish (Thermo Scientific, USA) UHPLC coupled to a Themo Q Exactive Focus MS was used and the SRM transitions of 4813 → 421 (product) and 4833 → 423 (internal standard) were monitored. Specific activities were calculated from the amounts of cystathionine produced and the protein contents of the samples.

### 4.7. Statistical Analysis

To assess significance, the two-sample *t* test was used and to interpret the relationship between the values correlation matrices were employed. Significance level was set at *p* < 0.05.

## 5. Conclusions

In conclusion, overload- induced hypertrophy of skeletal muscle is associated with increased SIRT1, Akt, mTOR, S6, and suppressed sestrin 2 levels, which are mostly responsible for anabolic signaling. On the other hand, the decreased FOXO1, and SIRT3 signaling suggest downregulation of protein breakdown and mitophagy. The decreased levels of NAD^+^, sestrin2, OGG1 indicate that the redox milieu of skeletal muscle after 14 days of overloading is reduced. This paper confirms that SIRT1 is involved in hypertrophy of skeletal muscle, and a causative relationship between SIRT1 and anabolic and catabolic signaling pathways was established. This study revealed new members of signaling pathways which have an active role in overload induced-hypertrophy of skeletal muscle. Some potential signaling agents, like H_2_S was excluded from the contributing molecules of overload induced-hypertrophy.

## Figures and Tables

**Table 1:** Gradient elution profile of H_2_S measurement with monobromobimane using 0.1% TFA/H_2_O (Solvent A) and 0.1% TFA/ACN (Solvent B).

Time (min)	Solvent B%
0	15
4762	35
14,178	35
17,328	90
18,904	90
20,479	15
23,629	15

## Data Availability

Not applicable.
